# Helminthiasis and Hygiene Conditions of Schools in Ikenne, Ogun State, Nigeria

**DOI:** 10.1371/journal.pntd.0000146

**Published:** 2008-01-30

**Authors:** Uwem Friday Ekpo, Simon Nnayere Odoemene, Chiedu Felix Mafiana, Sammy Olufemi Sam-Wobo

**Affiliations:** Department of Biological Sciences, University of Agriculture, Abeokuta, Nigeria; University of Kelaniya, Sri Lanka

## Abstract

**Background:**

A study of the helminth infection status of primary-school children and the hygiene condition of schools in Ikenne Local Government Area of Ogun State, Nigeria was undertaken between November 2004 and February 2005 to help guide the development of a school-based health programme.

**Methods and Findings:**

Three primary schools were randomly selected: two government-owned schools (one urban and the other rural) and one urban private school. No rural private schools existed to survey. A total of 257 schoolchildren aged 4–15 y, of whom 146 (56.8%) were boys and 111 (43.2%) were girls, took part in the survey. A child survey form, which included columns for name, age, sex, and class level, was used in concert with examination of stool samples for eggs of intestinal helminths. A school survey form was used to assess the conditions of water supply, condition of latrines, presence of soap for handwashing, and presence of garbage around the school compound. The demographic data showed that the number of schoolchildren gradually decreased as their ages increased in all three schools. The sex ratio was proportional in the urban school until primary level 3, after which the number of female pupils gradually decreased, whereas in the private school, sexes were proportionally distributed even in higher classes. The prevalence of helminth infection was 54.9% of schoolchildren in the urban government school, 63.5% in the rural government school, and 28.4% in the urban private school. *Ascaris lumbricoides* was the most prevalent species, followed by *Trichuris trichiura*, *Taenia* species, and hookworm in the three schools. Prevalence of infection in the government-owned schools was significantly higher than in the private school (*χ* ^2^ = 18.85, df = 2, *p*<0.0005). A survey of hygiene conditions in the three schools indicated that in the two government schools tapwater was unavailable, sanitation of latrines was poor, handwashing soap was unavailable, and garbage was present around school compounds. In the private school, in contrast, all hygiene indices were satisfactory.

**Conclusions:**

These results indicate that burden of parasite infections and poor sanitary conditions are of greater public health importance in government-owned schools than in privately owned schools. School health programmes in government-owned schools, including deworming, health education, and improvement of hygiene conditions are recommended.

## Author Summary

We studied intestinal helminth infection status in primary-school children and the hygiene conditions of primary schools in Ikenne Local Government Area of Ogun State, Nigeria in order to help guide the development of school-based health programmes. Two government-owned schools (one urban and the other rural) plus one privately owned school participated in the study. Demographic data from 257 children showed that the number of schoolchildren gradually decreased as their ages increased in all the three schools. The sex ratio was proportional in the urban school until primary level three, after which the number of female pupils gradually decreased, whereas in the private school, sexes were proportionally distributed even in higher classes. The prevalence of helminth infection in 232 of the children, however, was 54.9%, 63.5%, and 28.4% in the urban government, rural government, and private school, respectively. *Ascaris lumbricoides* was the most common parasite, followed by *Trichuris trichiura*, *Taenia* species, and hookworm in the three schools. Infection in the government-owned schools was significantly higher than in the private school (*p*<0.0005). The hygiene condition in the three schools indicated that water supply and sanitation were poorer in government schools than in the private school. We therefore propose that school health programmes such as deworming, health education, and improvement of hygiene conditions, be undertaken in government-owned schools.

## Introduction

It is estimated that more than one billion of the world's population is chronically infected with soil-transmitted helminths, and 200 million are infected with schistosomiasis [1]. The high prevalence of these infections is closely correlated with poverty, poor environmental hygiene, and impoverished health services [1],[2]. Parasitic helminths are known causes of morbidities such as nutritional deficiency [3], impaired physical development and learning ability [4], and socioeconomic deprivations in populations living in the tropics where poor hygiene conditions provide an optimal environment for their development and transmission [5]–[7]. In many parts of the developing world, children are reported to have an intestinal helminth infection prevalence rate ranging between 50% and 80% [8],[9]. Although several studies indicate that intestinal helminth infections are highly prevalent among schoolchildren in Ogun State, Nigeria [10]–[13], there is no reported statewide prevalence for intestinal helminths except for ascariasis [13]. Also there are no available data about the demography and hygiene conditions of the state's schools to help guide the development of school health programmes, which are a requirement for sustainable control of soil-transmitted helminths in schoolchildren [14].

At present there is no National School–based parasite or soil-transmitted helminth control programme in Nigeria. In the past, there have been sporadic and uncoordinated deworming programmes untaken by government officials without any baseline information or data. The present study has three aims: first, to evaluate demographic features and intestinal helminth infections among schoolchildren; second, to investigate hygiene conditions in schools; and third, to identify factors that are essential in the development of sustainable school health programmes.

## Materials and Methods

### Study area

Ikenne Local Government Area (LGA) is one of the twenty LGAs in Ogun State, Nigeria. It is highly urbanized and popular due to the fact that many influential citizens are from Ikenne. The LGA lies in the rainforest vegetation belt of Nigeria. The local government is made up of three urban towns and two rural villages. The urban towns are Iperu, Ikenne, and Ilisan, while the rural villages are Irolu and Ogere. There are 20 government-owned primary schools and 25 approved private primary schools in this LGA. The inhabitants of the Ikenne LGA engage in various types of businesses and for water depend mainly on boreholes, taps, and rainwater stored in tanks, as there are few rivers and streams in the study area.

### Selection of schools for baseline surveys

The study was carried out between November 2004 and February 2005. Three primary schools were chosen: A.U.D. Primary School, Irolu, is rural and government-owned; Salvation Army Primary School, Iperu, is urban and government-owned; and El-Shaddia Nursery and Primary School, Ilisan, is urban and private. These schools were randomly selected in the study area after stratifying to represent different ownership and predominant socieconomic status of location. There were no private schools located in rural area for inclusion in the study. In each school, all pupils were enrolled into the study so that their sociodemographic status could be determined.

### Consent and ethical approval

At the beginning of the study, the reason for the surveys and procedure for stool sample collection were explained to the children. A leaflet was distributed to the children to inform their parents or guardians about the nature of the study and to obtain their consent for their wards to participate in the study. Only pupils who returned a consent form signed by their parents/guardians were allowed to participate in the study. The study was approved by the Institutional Ethical Review Board of the University of Agriculture, Abeokuta, Nigeria.

### Surveys and sampling

Two survey forms were used. The student survey form included columns for each child's name, sex, age, school class level, and parasitic infection status [1]. For each school the students were divided into four groups according to their ages (4–6 y, 7–9 y, 10–12 y, and 13–15 y) to analyze their demographic characteristics. The school survey form was used to collect information on the schools' sanitation conditions, specifically: type of water supply, condition and type of latrines, availability of soap for handwashing, and presence of garbage piles around the school compounds.

A plastic container marked with identification number and the name of child was distributed to each pupil. One stool sample was collected from each pupil. Stool samples were examined within 12 h by the cellophane thick smear method for eggs of intestinal helminths [15], and the results were recorded on the corresponding student survey form. However, the study could not determine intensity of infection, because materials needed to perform such assays (such as stool templates for Kato-Katz quantitative test), were not available in the country for purchase.

### Statistical analysis

Differences in prevalence of intestinal helminths infection between age group, sex and school ownership and locations, were tested by chi-squared tests.

## Results

### Demography

**Figure 1:**
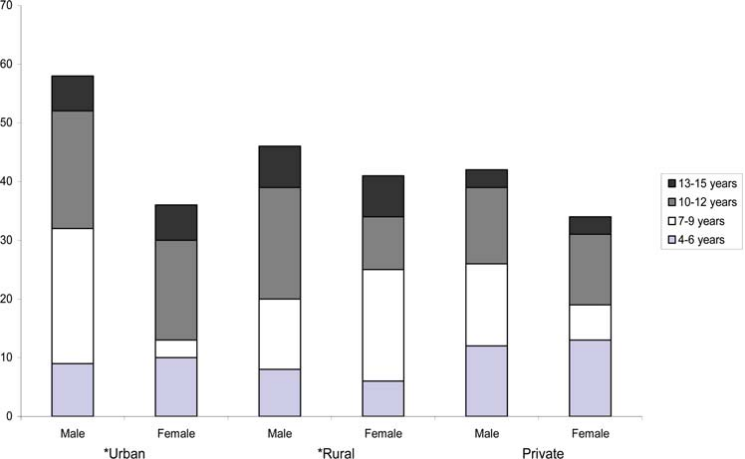
Demographic Characteristics of Schoolchildren in Government-Owned Urban and Rural Schools and an Urban Private School, Ikenne Local Government Area, Ogun State, Nigeria. The pair of bars on the right show age frequency data grouped by sex for an urban privately owned school. *The pair of bars on the left and in the center show data from an urban and a rural government school, respectively.

A total of 257 schoolchildren attending the three schools were analyzed for demographic characteristic (Figure 1). The age distribution was not proportional in the study area, as the number of children was decreasing as their age's increases with progress in school. Of the 257 students total, 146 (56.8%) were boys and 111 (43.2%) were girls. The sex ratio by class level was proportional until primary level 3, after which the number of females gradually decreased in the urban government school, while in the rural government and the urban private schools, schoolchildren were proportionally distributed between sexes even in the higher class levels (Figure 1).

### Hygiene conditions of schools

The hygiene condition of the three schools differed in several ways. Available water supply, presence of garbage around school compounds, conditions of latrines, and presence of soap in classrooms were different in the three schools (Table 1). These conditions were poorer in government-owned schools than in the private school. The water supply was inadequate in both government-owned schools; tap water was not constant, mostly twice a week and at times once a week in urban and rural government schools, respectively. Pupils brought bottle of water to school from their homes. In the private school, the water supply was regular, from a borehole. Pupils drank water using personal cups from the containers in their classes.

**Table 1:** Description of Hygiene Condition Indicators in the Three Schools.

Indicators	Government-Owned		Privately Owned
	Rural	Urban	
Year of establishment	1923	1932	2000
Condition of water supply	Irregular once a week	Irregular twice a week	Regular through a school borehole
Usage of water cups	Common cups	Common cups	Personal cups
Types of toilets facility	Pit latrine	Pit latrine	Water closet system
Number of toilets	Four latrines with four holes	Four latrines with four holes	Two water closet systems
Adequacy of toilets for school population	Inadequate	Inadequate	Adequate
Condition of toilet	Dirty	Dirty	Clean
Latrine lid	Absent	Absent	Not applicable
Usage of toilet	Abandoned	Abandoned	In use
Soap on washing basin in classroom	Absent	Absent	Not applicable
Condition of classrooms	Dirty	Dirty	Clean
Garbage cans in school	Absent	Absent	Present
Garbage around school compound	Present	Present	Present only in classrooms
Food vendors	Present	Present	Present
Types of food	Rice, beans, and stew; roasted meat (suya)	Meat pie; donuts (snacks); rice, beans, and stew; roasted meat (suya)	Meat pie; donuts (snacks); rice, beans, and stew; roasted meat (suya)
Food vending materials	Leaves	Plastic plates	Plastic plates
Food storage materials	Basins	Plastic warmers	Plastic warmers

The toilet facilities in government-owned schools were in poor condition. The government-owned schools are equipped with pit latrines constructed through contracts. In each government-owned school, there were four pit latrines: two for boys, one for girls, and one for the teachers. The pit latrines for the schoolchildren were so dirty that the pupils preferred to defecate in vegetation surrounding the school compounds; and there were no basin and soap for handwashing after using the toilet. In the private school, the toilet facility was a water closet system, cleaned by a cleaner employed by the school, and water was provided at regular intervals for flushing the toilets.

There were many garbage piles around the school compounds in the government-owned schools, and schoolchildren were seen playing and digging around the garbage. The garbage piles included mainly waste paper, food wrappings, industrial water sachets (50 cl polyethylene bags containing water), dry leaves, and other matter produced regularly both in classrooms and outside the classroom. Garbage cans were also absent in these schools. The private school was an exception (although some waste paper was found in the classrooms), and there was garbage can inside the school premises.

Food vendors were present in the three schools; in rural and urban government schools vendors served pupils in their classrooms, while in private schools most pupils brought their own food to school from home. Inspection of food vendors was not done in the three schools. Foods items served by vendors in the urban were snacks such as meat pies, donuts, and groundnuts (peanuts). In all schools vendors served local dishes such as rice, beans, stew, and roasted meat known as “suya.”

### Helminth infections

A total of 257 stool containers were distributed, and 232 (90.3%) were returned; 25 (9.7%) of the schoolchildren refused to submit a stool sample and therefore did not participate in the infection study. Compliance was 85.1% in the rural government school (A.U.D. Primary School, Irolu), 88.2% in the urban private school (El-Shaddai Nursery and Primary School, Ilisan), and 96.8% in the urban government school (Salvation Army Primary School, Iperu). Compliance was higher among lower-grade-level schoolchildren but decreased among higher-grade-level children in the the study area. In total, 232 schoolchildren were examined for intestinal helminth infection, and 116 (50.0%) were found to be infected with one or more helminths. The prevalence of helminth infections varied significantly (*χ* ^2^ = 18.85, df = 2, *p*<0.0005) among schoolchildren in the three schools. Prevalence rates were 63.5% in the rural government school, 54.9% in the urban government school, and 28.4% in the urban private school. Multiple infections were higher in the rural government school than in the urban government school, and no multiple infections were found in the private school. *Ascaris lumbicoides* was the most common infecting species in the three schools, while the prevalence of *Trichuris trichiura* was higher among schoolchildren in both rural and urban government schools than in the urban private school. The prevalence of *Taenia* species was higher in the rural school than in the urban government and private schools. Hookworm infection was low among schoolchildren in the urban and rural government schools, and there were none found in the private school (Table 2).

**Table 2:** The Prevalence of Intestinal Helminth Infections in Government-Owned (Urban and Rural) and Privately Owned (Urban) Schools.

Parasites	Government-Owned						Privately Owned Urban			*p*-Value
	Urban			Rural						
	No. Examined	No. Infected	%	No. Examined	No. Infected	%	No. Examined	No. Infected	%	
Any infection	91	50	54.9	74	47	63.5	67	19	28.4	<0.0005
Multiple helminth infection	91	11	12.1	74	15	20.3	67	0	0.0	0.001
*Ascaris lumbricoides*	91	34	37.4	74	29	39.2	67	11	16.4	0.005
*Trichuris trichiuria*	91	15	16.5	74	18	24.3	67	5	7.5	0.03
*Taenia* species	91	7	7.7	74	12	16.2	67	4	6.0	0.08
Hookworm	91	5	5.5	74	3	4.1	67	0	0.0	0.164

The prevalence of infection was further analyzed according to the age and sex of schoolchildren (Table 3). There were no significant differences in prevalence of infection between age groups of schoolchildren in the study schools. There were also no significant differences in infection between male and female schoolchildren in rural government and urban private school, but significantly more males were infected than females in the urban government school (*p* = 0.037) (Table 3).

**Table 3:** The Prevalence of Intestinal Helminth Infections by Age Group and Sex of Schoolchildren in the Government-Owned (Urban and Rural) and Private (Urban) Schools.

Category	Group	Government-Owned						Privately Owned		
		Urban			Rural					
		No. examined	No. infected	%	No. examined	No. infected	%	No. examined	No. infected	%
Age	4–6	13	9	69.2	6	5	83.3	25	5	20.0
	7–9	37	17	45.9	30	18	60.0	18	7	38.9
	10–12	31	18	58.1	26	17	65.4	20	6	30.0
	13–15	10	6	60.0	12	7	58.3	4	1	25.0
	*p*-Value	0.474			0.716			0.595		
Sex	Male	49	32	65.3	37	21	56.8	36	9	25.0
	Female	42	18	42.9	37	26	70.3	31	10	32.3
	*p*-Value	0.037			0.334			0.592		

## Discussion

This survey investigated demography, helminthiasis, and sanitary conditions in three primary schools of different ownership and social settings in Ikenne LGA of Ogun State, Nigeria. The study shows clearly that the burden of parasitic infections in schoolchildren and poor sanitary conditions of the urban and rural schools owned by the government constitute a public health priority. It strongly supports the need for school health programmes aimed at reducing the prevalence of helminth infections in schoolchildren and improving the sanitation conditions in and around the schools. The demographic data, however, indicated that the proportion of schoolchildren benefiting from a school health programme would decline with increasing grade-level and that female children would be increasingly disadvantaged in government-owned schools.

The demographic data indicate that the number of schoolchildren gradually decrease with increasing age in government-owned urban and rural schools. This pattern may be due to a high rate of dropouts occasioned by lack of funds and/or parents sending their children to learn handwork/craftwork rather than to complete primary education. Another point revealed by the demographic data is that fewer female than male children attend government-owned urban and rural schools. Several socioeconomic and behavioural reasons may be involved in the female dropout rate [14], but it is probably due to three main reasons in this locality. First, parents considered it a waste of resources to invest in education of girls, and/or that educating a girl is a waste of resources as they will eventually be married out of the family. Second, some parents might believe that female children should stop attending school at puberty in order to avoid unwanted pregnancies. Third, in many parts of Africa, including Nigeria, female children are withdrawn from schools to be engaged as domestic house helpers or child labourers, especially in polygamous families. However, parents sending their wards to the private school give female children equal educational opportunity up to high-grade education. Higher socioeconomic and educational status of the parents may explain the attitude that female children should have opportunities to gain skills and capabilities equal to those available to male children. These demographic features are also similar among schoolchildren surveyed in Chad, Mali, Ghana, and Tanzania in Africa [16],[17], and in Turkey [18]. It is hoped that federal legislation making primary education free and compulsory in Nigeria will increase the level of enrolment of girls.

It is suggested that school health programmes will also offer the potential to attract children to school, to receive treatment and other benefits. These programmes may be in the form of school health clubs, deworming programmes, and teaching of basic hygiene education subjects. Such programmes must be participatory in both design and implementation, and involve government health departments, school boards, teachers, and the pupils themselves. The programme should also incorporate a process to reward best-performing schools in hygiene standards and to encourage competition among schools.

It is well known that, due to the falling standard of teaching in government schools, many parents/guardians prefer to send their wards to private schools, even at a very high, burdensome cost to them. Government resources to run and manage public schools over the years have been hampered through corruption and mismanagement of funds. Therefore, there are limited resources to improve the quality of education in government primary schools. However, parents still send their ward to public schools, because it is free.

This study indicates that prevalence of intestinal helminth infection was higher in government-owned urban and rural schools than in the urban private school. This result was expected, because the poor socioeconomic status, poor hygienic habits, and lack of sanitation in these settings all support helminth infection, as suggested by previous studies [19],[20]. In the government schools, water supplies were insufficient (deliveries one or twice in a week), and the toilet facilities—which were dilapidated latrines—were unsanitary. There was no soap for handwashing after using the latrine. Because of the condition of the latrines, most schoolchildren defecated around the school compounds and did not clean their hands afterward because of the lack of both water and education about good hygiene.

Garbage piles were accumulated around government-owned schools, and schoolchildren were seen digging and playing on them. Thus, the children may have been exposed to an additional risk for the transmission of infection in the rural and urban school, which may explain the higher prevalence of intestinal worm infections. Regularly emptied garbage cans are needed in public schools.

Food vendors are characteristic of many public schools in developing countries, as they provide snacks and lunches to pupils. However, their sources of food and mode of preparations have always been a source of concern to school authorities, who often try to ban them from their premises. However, in the absence of alternative sources of food for the children, it is suggested that school feeding programmes that utilize reliable food sources be encouraged. Schoolchildren in the urban private school also suffered a notable infection rate (28.4%), although this is substantially lower than in the government urban school at 54.9% and rural school at 63.5%; Table 2), although their school was more sanitary and the parents were in better socioeconomic conditions.

This study reconfirms that the three most common intestinal helminths are *A. lumbricoides, T. trichiura,* and hookworm as documented previously [6]. These three species are cosmopolitan; *Ascaris* spp. and *Trichuris* spp. are transmitted by the faecal-oral route, while hookworms active penetrate exposed skin. Presence of ascariasis and trichiuriasis indicates that food and water are contaminated with infective eggs of these parasites by any of a number of routes, or that hand-to-mouth transmission may occur. Food and drinking-water handling equipment may be contaminated if there are no safe and secured human waste disposal methods or handwashing facilities—as is the case in government-owned schools where pupils defecate around school compounds and are unable to wash their hands because there is no soap and only infrequent water. The hookworm infections observed may have been acquired by children who do not wear protective shoes, which is very common among schoolchildren in government-owned schools. Students in these schools usually remove their shoes when they are playing within and outside school premises, because they may have a single pair of shoes for a whole session and need to make them last. Taeniasis could have been acquired from consumption of raw or improperly cooked meat (beef and pork) in the form of a locally roasted delicacy called “suya.” This improperly cooked meat is usually provided by food vendors on exposed trays.

Because of the known devastating effects of these parasite infections on the physical and mental conditions of children, it is suggested that a control programme against these infection commence as soon as possible. Such a programme should adopt the use of combined interventions. One such intervention is periodic deworming of children, at least once every year, using information based on infection rate, intensity [1], and reinfection studies, particularly for ascariasis [21]. Another is the provision and use of basic amenities and health education on the dangers, modes of transmission, and prevention of these intestinal parasites. Yet another is to address the problem of poor water supply and poor sanitation conditions in government schools—this measure would help a deworming programme succeed. Finally, local health officials and school management should collaborate to initiate school health programmes for delivering anthelmitic drugs and health education activities to these schools.

The results of this study have provided baseline information for planning school-based health education programmes in the rural and urban government-owned schools studied. Additional funds will be needed to provide the same information for other schools in Ikenne LGA in particular, and Ogun State in general.
